# The Relationship between Diabetes Mellitus and Respiratory Function in Patients Eligible for Coronary Artery Bypass Grafting

**DOI:** 10.3390/ijerph15050907

**Published:** 2018-05-03

**Authors:** Aleksandra Szylińska, Mariusz Listewnik, Żaneta Ciosek, Magdalena Ptak, Anna Mikołajczyk, Wioletta Pawlukowska, Iwona Rotter

**Affiliations:** 1 Department of Medical Rehabilitation and Clinical Physiotherapy, Pomeranian Medical University, 70-210 Szczecin, Poland; ciosekzaneta@gmail.com (Ż.C.); ptak.magda@gmail.com (M.P.); mikolajczyk.ania1@wp.pl (A.M.); wsna@o2.pl (W.P.); iwrot@wp.pl (I.R.); 2 Department of Cardiac Surgery, Pomeranian Medical University, 70-111 Szczecin, Poland; sindbaad@poczta.onet.pl

**Keywords:** spirometry, diabetes mellitus, cardiac surgery

## Abstract

*Introduction:* Spirometry performed prior to surgery provides information on the types of lung disorders in patients. The purpose of this study was to look for a relationship between the prevalence of diabetes and spirometry parameters. *Material and Methods:* The study was conducted in patients with coronary artery disease who were eligible for an isolated coronary artery bypass graft in 2013. The study group included 367 patients (287 men and 80 women) aged 68.7 ± 8.4 years. They were divided into those with diagnosed diabetes (group I, *n* = 138, 37.6%) and without diabetes (group II, *n* = 229, 62.4%). Spirometry tests were performed on the day of admission to the hospital. *Results:* Patients with diabetes (group I) had a significantly higher body mass index than those without diabetes (group II). Spirometry tests also showed that patients with diabetes had statistically significantly lower forced vital capacity (FVC) and forced expiratory volume in 1 s (FEV1.0). Both FVC and FEV1.0 were also statistically significantly lower for overweight and obese individuals in group I than those in group II. *Conclusion:* Patients with diabetes eligible for coronary artery bypass grafting with concurrent overweight or obesity are more likely to have lower spirometry parameters than those without diabetes.

## 1. Introduction

Similar to other industrialized countries, Poland has seen an ever-increasing incidence of diabetes. The growing proportion of patients with diabetes has also been observed in the population of patients with ischemic heart disease. As indicated by all large randomized studies, the most effective method of revascularization of the myocardium in this increased risk group (i.e., concomitant ischemic heart disease and diabetes) is coronary artery bypass grafting (CABG), a key treatment for advanced ischemic heart disease [1]. 

CABG is performed by median sternotomy, significantly affecting the mobility of the chest. Prior to the operation, pulmonary function and capacity can be assessed with the use of spirometry, which measures air volume in the lungs and airflow through the airways during inhalation and exhalation [2,3]. Spirometry is used as an important test for diagnosis, management, and evaluation of treatment, which in part enables prediction of the development of multiple respiratory diseases [4]. It provides information about lung function disorders prior to surgery, and helps in the prediction of improvements in respiratory parameters [5].

Diabetes is a chronic metabolic disease caused by a disorder of insulin secretion. According to the International Diabetes Federation (IDF), about 3.1 million people in Poland suffered from diabetes in 2011 [6]. Diabetes is a multisystemic disease that is primarily associated with the pancreas, but also affects the skeletal muscles, gastrointestinal tract, kidneys, and brain [7]. It is increasingly observed in people diagnosed with heart disease [8]. In diabetics, the respiratory system is characterized by a growth of fibroblasts and vascular endothelium, increased density of pulmonary microspheres, and thickening of the walls of pulmonary vesicles caused by increased collagen and elastin [9]. These related types of damage to the alveolar–capillary barrier and reduction in pulmonary capacity and flow result in further adverse effects on the functional state of the body. This may not only contribute to deepening respiratory failure symptoms, but also indirectly affect the cardiovascular system [10]. The association of these changes with diabetes has been demonstrated in both animal studies and human autopsy studies [11]. 

The purpose of the study was to investigate the potential relationships between the occurrence of diabetes and respiratory parameters.

## 2. Material and Methods

### 2.1. Participants

The study was carried out in 2013 and began with a group of 502 patients with coronary artery disease who were eligible for coronary artery bypass grafting at the Department of Cardiothoracic Surgery of the Pomeranian Medical University in Szczecin. Patients who had not had a spirometry test were excluded from the study. Also excluded were patients with a recent myocardial infarction, with acute coronary syndrome, or with resting pains, those who could not be reliably examined, and those who refused to participate in the study. Further analysis involved 367 patients (287 men and 80 women) aged 68.7 ± 8.4 years. All patients had already had their first isolated coronary bypass surgery with extracorporeal circulation. All patient provided written informed consent. The study was approved by the Bioethics Committee of Pomeranian Medical University in Szczecin (KB-0012/121/14).

The first group consisted of 138 patients with diagnosed diabetes (37.6%), while the second group consisted of 229 patients without diabetes (62.4%). 

### 2.2. Data Collection

On the day of admission to the hospital, each patient was subjected to a spirometry test performed with an AsSPIRO D200 v.101 (Aspel, Zabierzów, Poland). The study was conducted according to the recommendations of the American Thoracic Society (ATS) and the European Respiratory Society (ERS) [12,13,14,15]. The following parameters were evaluated: forced vital capacity (FVC), forced expiratory volume in one second (FEV1), and peak expiratory flow (PEF).

The following anthropometric measurements were made: blood pressure, body mass, and body height. Interviews were conducted to determine the concurrent presence of hypertension, renal disease, neoplasms, neurological diseases, pulmonary diseases, and other cardiovascular diseases. Body mass index (BMI) was calculated using the formula BMI = body weight (kg)/height (m)^2^. Normal body weight was diagnosed for BMI in the range 18.5–24.99 kg/m^2^, overweight for 25–29.99 kg/m^2^, and obesity for ≥30 kg/m^2^. 

### 2.3. Statistical Analysis

Statistical analysis was performed with the use of Statistica v12 software (StatSoft, Inc., Tulsa, OK, USA). The normality of distribution was assessed using a Shapiro-Wilk test.

Due to the lack of normal distribution, further analyses were based on the Mann-Whitney U or Kruskall-Wallis test. Chi-square test was used for qualitative data. Qualitative variables were expressed as percentage (%). Logistic regression models were used to determine odds ratios and confidence intervals. Three individual models for diabetes were used to characterize the relationships between diabetes and the spirometry parameters FVC, FEV1.0, and PEF. Three subsequent models showing the relationship between diabetes and spirometry parameters were adjusted for gender, age, BMI, and cigarette smoking. Discriminant analysis was used. The significance level was set at *p* ≤ 0.05.

## 3. Results 

In the spirometry tests, patients with diabetes had significantly lower FVC and FEV1.0 levels than those without diabetes, while mean peak expiratory flow measurements were not significantly different. The EuroSCORE logistic’s operational risk ratio was significantly higher in patients with diabetes. The data is shown in Table 1.

Body mass index was significantly higher in patients with diabetes. Patients with diabetes had the highest proportion of obesity (58.7%, *n* = 81) and the lowest proportion of correct body weight (5.07%, *n* = 7) (*p* < 0.001).

Compared to the patients with diabetes, there was a greater proportion of overweight people among those without diabetes (49.8%, *n* = 114), and a higher proportion of patients with normal weight (18.34%, *n* = 42; *p* < 0.001). An ejection fraction above 50% was found in 52.54% patients with diabetes (*n* = 62) and 65.32% patients without diabetes (*n* = 162; *p* = 0.02). There were 18 patients (14.78%) with diabetes and 17 patients (7.82%) without diabetes (*p* = 0.03) in the high-risk euroSCORE group (8 points). Eight patients (5.8%) with diabetes and 16 patients (6.99%) without diabetes had chronic obstructive pulmonary disease, although the differences in the groups were not statistically significant.

**Figure 1:**
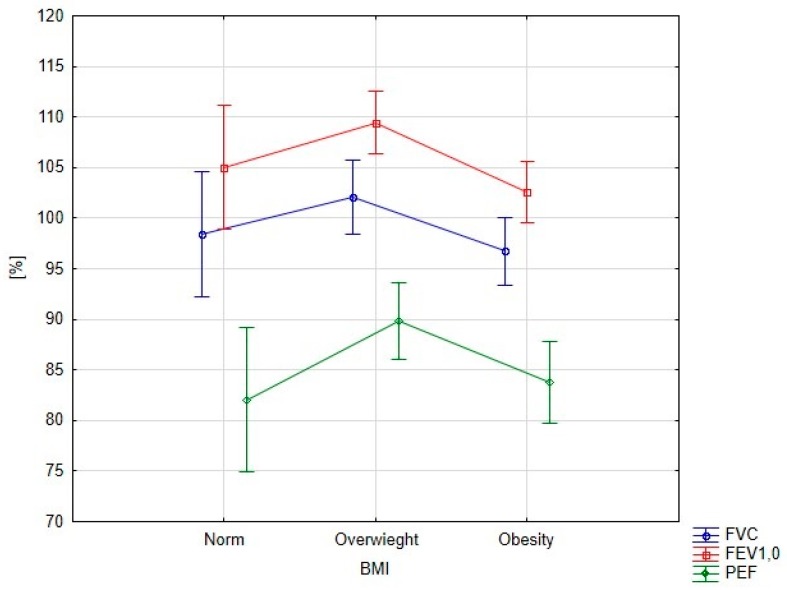
Spirometry parameters depending on body mass index (BMI).

Significant differences in spirometry parameters depending on BMI were found for FEV1.0 (*p* = 0.008) and PEF (*p* = 0.042) (Figure 1). All overweight patients exhibited higher spirometry parameters than patients with obesity or normal weight.

**Figure 2:**
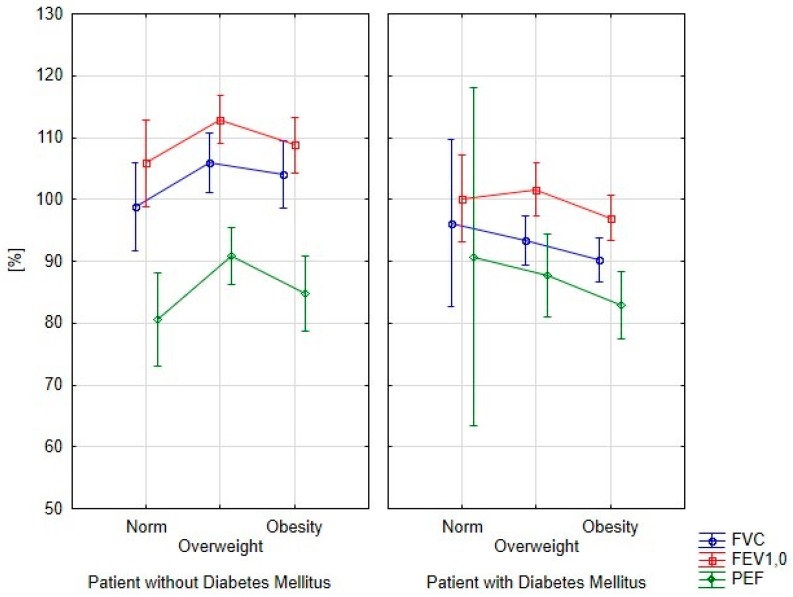
Spirometry parameters depending on BMI in patients with and without diabetes mellitus.

An analysis of spirometry parameters depending on BMI is shown in Figure 2. Obese patients with diabetes had reduced levels of spirometry parameters. However, differences in the analyzed parameters were not statistically significant, similar to the group without diabetes. Significantly, the lowest spirometry parameters in patients without diabetes were obtained in patients with normal weight.

Multivariate logistic regression analysis is presented in Table 2. The column with nonadjusted values shows each spirometry parameter that was individually entered into the model. In the second column, each spirometry parameter is separately adjusted for gender, age, BMI, and cigarette smoking.

There was a similar effect of spirometry parameters with and without adjustments. In the model with adjustments, there was also a significant effect of BMI on diabetes. For the forced vital capacity model, the odds ratio for BMI was 1.169 (*p* < 0.001). In the model with forced expiratory volume in one second, the odds ratio for BMI was 1.163 (*p* < 0.001), and in the PEF model the odds ratio for BMI was 1.178 (*p* < 0.001). 

## 4. Discussion

For many years, researchers have discussed the effects of diabetes on the respiratory system [9]. In this study, patients with diabetes eligible for coronary artery bypass grafting had significantly reduced FVC and FEV1.0 compared to patients without diabetes. This is consistent with the results reported by other authors [16,17,18,19,20]. Our research shows a decrease in spirometric parameters with and without adjustment, which indicates that demographic data did not interfere with the decrease in FVC and FEV1.0 in patients with diabetes.

In a long-term follow-up study of patients with diabetes, Kaminsky observed a gradual decline in FVC and FEV1.0 [17]. Similarly, in a seven-year follow-up, Davis et al. demonstrated a decrease in FEV1.0 in patients with diabetes, and, in addition, by analyzing the risk of death, they found that a 10% decrease in FEV1.0 could contribute to increased mortality [18]. In a study conducted by Litonjua et al. on 352 men with diabetes or at risk for diabetes, spirometry parameters, mainly FVC and FEV1.0, were lower compared to those of 352 healthy subjects [19]. Similar results were obtained in a three-year assessment by Yeh et al. in which 1100 patients with diabetes had lower FVC and FEV 1.0 than those without diabetes. After three years the results deteriorated further and the differences were statistically significant [20]. Lawlor et al. also demonstrated a reverse correlation between FVC and FEV1.0 and type 2 diabetes in women [21]. Similar results were obtained in our study, as patients with diabetes eligible for CABG had lower FVC and FEV1.0 compared to those without diabetes.

While forced vital capacity is a static parameter that is difficult to compare unless adjusted for patient gender, age, and body weight, forced expiratory volume in one second is a functional parameter that reflects the volume and flow of air through the airways, and as such is more useful [22]. McAllister et al. described FEV1.0 as a strong predictor of high postoperative mortality rate and longer hospital stay in cardiac surgery patients. In addition, McAllister argues that FEV1.0 should be included in the current euroSCORE perioperative risk assessment [23].

It has been shown that obesity increases the risk of type 2 diabetes [24,25]. People with a BMI above 35 have a 20 times higher risk of developing diabetes than those with a BMI within the normal range [24]. The concurrence of diabetes and obesity significantly increases the risk of cardiovascular complications and mortality [26,27,28].

Every patient eligible for cardiac surgery should have a spirometry test prior to surgery to provide information about their respiratory status. It could significantly help in planning anesthesia, surgery, and postoperative care.

## 5. Conclusions

In this study, obese and overweight patients with diabetes eligible for CABG had lower spirometry parameters compared to patients without diabetes. This indicates that these categories of patients require respiratory tests and treatment aimed at improving respiratory function before and after cardiac surgery.

## 6. Limitations

The limitation of our study consisted in the low number of patients with diabetes with normal BMI. As the majority of patients with diabetes had above-normal BMI, further tests with a larger number of patients with normal body mass would yield more reliable results. The relevance of the study is also limited by the fact that spirometry was not measured after surgery, which will be addressed in our future research.

## Figures and Tables

**Table 1:** Descriptive statistics of respiratory capacity in relation to the occurrence of diabetes mellitus.

Variable		Patients without Diabetes Mellitus (*n* = 229)	Patients with Diabetes Mellitus (*n* = 138)	*p*
Sex (*n*, %)	Female	46 (20.09%)	34 (24.64%)	0.306
	Male	183 (79.91%)	104 (75.36%)	
Age (years)		68.13 ± 8.55	68.91 ± 7.56	0.407
BMI (kg/m^2^)		28.26 ± 4.12	30.84 ± 3.96	<0.001 *
Smoking (*n*, %)	Yes	100 (43.67%)	55 (39.86%)	0.473
	No	129 (56.33%)	83 (60.14%)	
Smoking (years)		35.63 ± 10.94	32.08 ± 10.49	0.118
Case priority (*n*, %)	Planned	182 (80.53%)	110 (80.88%)	0.934
	Urgent and emergent	44 (19.47%)	26 (19.12%)	
ESL (%)		3.76 ± 3.38	4.68 ± 5.17	0.119
EF (%)		49.56 ± 9.32	48.22 ± 9.98	0.132
Concomitant diseases				
COPD (*n*, %)	Yes	16 (6.99%)	8 (5.80%)	0.655
	No	213 (93.01%)	130 (94.20%)	
Stroke (*n*, %)	Yes	15 (6.55%)	13 (9.42%)	0.316
	No	214 (93.45%)	125 (90.58%)	
Chronic renal failure (*n*, %)	Yes	8 (3.49%)	9 (6.52%)	0.181
	No	221 (96.51%)	129 (93.48%)	
Arterial hypertension (*n*, %)	Yes	174 (75.98%)	115 (83.33%)	0.095
	No	55 (24.02%)	23 (16.67%)	
Spirometry test	FVC (%)	103.97 ± 24.61	91.65 ± 15.45	<0.001 *
	FEV1.0 (%)	110.30 ± 20.99	98.82 ± 15.70	<0.001 *
	PEF (%)	87.00 ± 25.26	84.97 ± 24.50	0.771

BMI, body mass index; ESL, euroSCORE logistic; EF%, ejection fraction; COPD, chronic obstructive pulmonary disease; FVC, forced vital capacity; FEV1.0, forced expiratory volume in 1 s; PEF, peak expiratory flow; *p*, statistical significance; *n*, group size; * Statically significant parameter.

**Table 2:** Univariate regression analysis for patients with diabetes.

Variable	Nonadjusted				Adjusted ^a^			
	OR	−95% CI	+95% CI	*p*	OR	−95% CI	+95% CI	*p*
FVC (%)	0.971	0.959	0.982	<0.001 *	0.969	0.957	0.981	<0.001 *
FEV1.0 (%)	0.968	0.955	0.980	<0.001 *	0.966	0.953	0.980	<0.001 *
PEF (%)	0.997	0.988	1.005	0.449	0.998	0.988	1.008	0.734

*p*, statistical significance; *n*, group size; FVC, forced vital capacity; FEV1.0, forced expiratory volume in 1 s; PEF, peak expiratory flow; OR, odds ratio; CI, confidence interval. ^a^ Adjusted for gender, age, BMI, cigarette smoking; * statically significant parameter.
